# Evaluating Human Intestinal Cell Lines for Studying Dietary Protein Absorption

**DOI:** 10.3390/nu10030322

**Published:** 2018-03-07

**Authors:** Paulus G. M. Jochems, Johan Garssen, Antonius M. van Keulen, Rosalinde Masereeuw, Prescilla V. Jeurink

**Affiliations:** 1 Division of Pharmacology, Utrecht Institute of Pharmaceutical Sciences, Utrecht University, 3584 CG Utrecht, The Netherlands; p.g.m.jochems@uu.nl (P.G.M.J.); j.garssen@uu.nl (J.G.); a.m.vankeulen@students.uu.nl (A.M.v.K.); p.v.jeurink@uu.nl (P.V.J.); 2 Nutricia Research, Nutricia Research, Globel Center of Excellence Immunology, 3584 CT Utrecht, The Netherlands

**Keywords:** peptides, amino acid, transport, Caco-2, HT-29, T84

## Abstract

With the global population rising, the need for sustainable and resource-efficiently produced proteins with nutritional and health promoting qualities has become urgent. Proteins are important macronutrients and are involved in most, if not all, biological processes in the human body. This review discusses these absorption mechanisms in the small intestine. To study intestinal transport and predict bioavailability, cell lines are widely applied as screening models and often concern Caco-2, HT-29, HT-29/MTX and T84 cells. Here, we provide an overview of the presence and activities of peptide- and amino acid transporters in these cell models. Further, inter-laboratory differences are discussed as well as the culture micro-environment, both of which may influence cell culture phenotype and performance. Finally, the value of new developments in the field, including culturing cells in 3-dimensional systems under shear stress (i.e., gut-on-chips), is highlighted. In particular, their suitability in screening novel food proteins and prediction of the nutritional quality needed for inclusion in the human diet of the future is addressed.

## 1. Introduction

Our diet primarily consists of a macronutrient mixture. Carbohydrates, lipids and proteins add up to 51.8%, 32.8% and 15.4%, respectively, of the total food-derived energy in the Western diet [1]. These numbers show that proteins resemble a relatively small amount of the total food energy compared to the other macronutrients, mainly because they are not primarily dedicated to energy delivery. However, proteins are involved in many, if not all, biological processes (e.g., cell growth and differentiation) and are frequently referred to as “the building blocks of the human body” [2,3]. According to the World Health Organization, the world population is projected to reach more than 9 billion by 2050 accompanied by an essential doubling of the food demand. This urges the need for sustainable and novel nutrients, such as proteins. Prior to human consumption, these novel proteins need to be studied and declared safe for consumption.

After consumption of food, the digestive process starts in the oral cavity. Within the mouth, chewing results in smaller pieces, and amylases start breaking down the carbohydrates [4]. However, the digestive process of proteins starts in the stomach [5]. During digestion, enzymes are secreted as zymogens, an enzymatically inactive form, by chief cells [2], which secrete pepsinogen upon stimulation by, e.g., gastrointestinal hormones [6]. These gastrointestinal hormones are secreted after ingestion of a meal. The amount that is secreted depends on meal composition and calories. The acidic environment denaturizes proteins and transforms the zymogen pepsinogen into its active form, pepsin. The gastric phase is followed by the small intestinal phase, consecutively passing through the duodenum, jejunum and ileum. The small intestine exists of a heterogeneous cell populations. Four major cell types can be distinguished, of which the absorptive enterocytes represent approximately 90% of the total number. The remaining 10% consists of mucus-producing goblet cells, enteroendocrine cells and antimicrobial peptide-producing Paneth cells [7]. The intestinal epithelium is covered by mucus produced by goblet cells. The mucus contains a high number of different enzymes, antimicrobial peptides, immune factors, growth factors and many more constituents. Mucins feed the microbiota and protect the epithelium from the microbiota, and slow down the diffusion rate of the nutrients [8,9]. Besides these four major cell types, there are immune players present such as macrophages, dendritic cells, microfold-cells (M-cells) and Peyer’s patches [10].

In the duodenum, the liver and pancreas secrete bile and pancreatic juice, respectively [11]. Bile is composed of bile salts (derivatives of cholesterol) and plays a major role in digestion. After emulsification, the lipids can be hydrolyzed within the micelle [2]. The pancreatic juice contains a mixture of bicarbonate and proteases which play an important role in protein digestion. Bicarbonate alkalizes the acidic food bolus coming from the stomach. The resulting neutral environment (pH = 6.5–7.5) is ideal for the activation of the proteases secreted by the pancreas [12]. These proteases are secreted as zymogens and are activated upon enzymatic cleavage [13]. The pancreatic proteases can be divided into endopeptidases (hydrolyzing peptides bonds in the interior of the amino acid chain) and exopeptidases (releasing the amino acids from either end of the amino acid chain). Next to these enzymes, the small intestinal brush border located at the epithelial apical membrane contains a number of brush border enzymes, among others, carboxypeptidase, aminopeptidase N, tripeptidase and dipeptidase [14]. These carboxypeptidase and aminopeptidase are able to completely digest a protein into a mixture of peptides and amino acids.

The duodenum is followed by the jejunum where the majority of absorption processes take place. Just before absorption, protein digestion is finalized by the microbiota and intestinal brush border harboring enzymes. The digestive products of proteins are di- and tripeptides and amino acids [15]. Compared to the large intestine, the small intestine harbors a relatively low density of microbiota due to the rapid flow and anti-microbial substances secreted by Paneth cells [16]. Peptides and amino acids can be taken up by the microbiota, depending on the bacteria’s transporter expressions. Once taken up, peptides and amino acids can be used for the synthesis of bacterial cell components or catabolized via other pathways. Their mode of action can either beneficially or detrimentally affect the host [17]. The microbiota also possess proteinases and peptidases, which might cooperate with the human proteases [18]. This review thoroughly discusses the absorption mechanisms of peptides and amino acids by the enterocytes. The ileum, the final part of the small intestine, completes nutrient and bile salts absorption. After this absorption, the eventual bioavailability is determined by effective transport over the basolateral membrane, allowing the nutrients to become systemically available [19]. The efficient transcellular transport of peptides occurs via cooporation between the apical and basolateral transporters [20].

The small intestine is followed by the large intestine, which harbors primarily a wide spectrum of microorganisms and reabsorbs liquids and salts. Absorption of food components in this segment is, however, limited and will not be taken into account in this review.

To study the intestinal absorption processes of proteins and their derivatives, human intestinal cell lines are commonly used. We here discuss human intestinal di- and tripeptide and amino acid transporter-mediated uptake and efflux, and the presence of these transporters in frequently used commercially available human intestinal cell lines. Additional attention is paid to their capability to mimic human intestinal absorption processes using advanced cell culturing methods. Furthermore, we will discuss innovations in intestinal in vitro models for studying human intestinal absorption.

## 2. Intestinal Absorption

The intestine offers four different routes of absorption: paracellular diffusion, transcellular passive diffusion, transcytosis and carrier-mediated transport [7,21]. Paracellular transport occurs through movement via the inter-cellular space, regulated by tight- and adherent junctions. Furthermore, tight junctions are important for the barrier function of the intestine, preventing hazardous compounds from freely passing through the intestinal wall. The intestinal permeability can be modulated by proteins and amino acids. For example, an arginine-rich protein decreases paracellular flow whereas L-alanine increases paracellular flow [22]. Thus, amino acids are capable of influencing paracellular flux. Transcellular passive diffusion is concentration-based and energy independent. Transcytosis is achieved via (receptor-mediated) endocytosis and involves compound transportation through the cell via unique vesicles (endosomes). The vesicles can be released at the basolateral side via exocytosis or digested within the lysosomes. Transporter-mediated transport is regulated via specific proteins in the cell membrane [21] that function via sym-, anti-, and uniporter mechanisms. Symporters translocate compounds via cotransport in the same direction over the plasma membrane, whereas antiporters transport compounds in opposite directions. Uniporters function unidirectional, without cotransport.

## 3. Intestinal Di- and Tripeptide and Amino Acid Transporters

Proteins differ in absorption rate, depending on multiple factors such as amino acid composition (side chains, groups at N- and C-terminus), protein origin and processing (e.g., heating) [23,24,25]. The transporters responsible for the uptake of di- and tripeptides and amino acids belong to the solute carrier (SLC) and cadherin (CDH) gene families.

Currently, four apical peptide transporters are known; *SLC15A1* (peptide transporter 1, PEPT1), *SLC15A3* (peptide histidine transporter 2, PHT2), *SLC15A4* (peptide histidine transporter 1, PHT1), are members of the peptide transporter family (PTR; *SLC15*) and *CDH17* (human peptide transporter 1, HPT-1) a member of the 7D cadherins family [26,27]. There is only one peptide transporter known to be present at the basolateral membrane, referred to as the basolateral peptide transporter. The encoding gene of the basolateral peptide transporter is, to our knowledge, unknown. PEPT1 is expressed in the human duodenum, jejunum and ileum and located at the brush border membrane [28]. Herrera-Ruiz et al. [27] showed the highest PEPT1 expression in the duodenum, decreasing in the jejunum and ileum and no expression in the colon. PHT2 and HPT-1 expressions were not region specific. Abidi et al. [29] investigated in vivo absorption of di-peptides and demonstrated that the jejunum showed the highest transport activity followed by the ileum and duodenum.

Free amino acids are absorbed in the small intestine, primarily in the proximal jejunum [24]. An overview of the diverse characteristics of the transport proteins present in the intestine is summarized in Table 1.

As most of the transporters depend on Na^+^, H^+^, Cl^−^ or K^+^, maintaining their ion gradients is of utmost importance. To prevent the loss of the proton gradient, the Na^+^/H^+^ exchanger 3 (NHE3), encoded by *SLC9A3*, plays a key role. NHE3 is an antiporter transporting Na^+^-ions into the cell in exchange for a proton [30]. Furthermore, ion-channels, ATPases and exchangers are involved in maintaining the desired ion gradients [31]. For the apical transporter system b^0,+^ to become active, dimer formation between rBAT and b^0,+^AT is essential. For the basolateral amino acid transporter systems y^+^L, L and Asc, formation of a dimer with the 4F2 cell surface antigen heavy chain (4F2hc) is needed. 4F2hc is encoded by *SLC3A2* and forms disulfide bonds with the amino acid transporter to direct it to the plasma membrane and assist in the proper assembly for the transporter to become active [32,33].

The transporter expression is regulated via different signaling pathways, involving the kinases general control nonderespressible 2/activating transcription factor 4 (GCN2/ATF4) and mammalian target of rapamycin (mTOR). These pathways are triggered via constant monitoring of the intracellular amino acid pool [29,34], where GCN2 and ATF4 are activated during amino acid starvation and mTOR upon amino acid abundance. These pathways have been described recently in more depth by Jewell et al. [35] and Taylor et al. [36]. Therefore, we do not elaborate on the intracellular regulation of amino acid transporters in this review.

## 4. In Vitro Models for Intestinal Peptide and Amino Acid Absorption

A representative human in vivo intestinal tract model should demonstrate the presence of a barrier, a brush border that produces enzymes, and heterogeneous cell populations and mechanical forces such as shear stress should be present. To study the adult absorption of di- and tripeptides and amino acids, intestinal cell lines are commonly used. ATCC offers a wide range of commercially available human intestinal cell lines: fetal small intestinal derived cell lines: HIEC-6 (7 Pubmed hits) and FHs 74 Int (33 Pubmed hits), adult duodenum adenocarcinoma derived: HUTU80 (21 Pubmed hits) and adult colon adenocarcinoma derived: Caco-2 (15,686 Pubmed hits), HT-29 (13,357 Pubmed hits) and T84 (1453 Pubmed hits). As Caco-2, HT-29 (including HT-29/MTX clone) and T84 are the most studied and characterized cell lines, we focus on these in this review. Each cell line has its own differentiation characteristics. Caco-2 cells are preferred to study absorption and transport processes, as these cells spontaneously differentiate into a small intestinal phenotype [43]. By contrast, HT-29 cells, cultured under standard conditions (viz. in the presence of glucose and (fetal calf) serum), do not form a tight barrier and are therefore not suitable for this purpose [44,45]. However, the HT-29 cells do show a goblet cell differentiation phenotype and produce mucus. Modifications in the standard culture conditions resulted in more enterocyte-like phenotypes and a more pronounced presence of the Goblet cell phenotype. Prolonged cultivation under modified culture conditions leads to the generation of clones. An example of a commonly used subclone is the HT-29/MTX. This subclone originated upon addition of methotrexate (1 µM) to the culture media and is capable of forming a tight monolayer and expressing relevant brush-border enzymes and is commonly used to study the role of goblet cells [46], whereas T84 cells are known for their crypt-like differentiation [47]. We now discuss the three cell models and a sub-clone that have been widely applied in amino acid, and di-and tripeptide transport studies and their characteristics.

## 5. Caco-2

### 5.1. Caco-2: Peptide Transporter Expression

Caco-2 cells express all relevant apical and basolateral intestinal peptide transporters. Herrera-Ruiz et al. [27] compared different parts of the human gastrointestinal tract with their Caco-2 cells and demonstrated that the cells expressed PEPT1, PHT1, PHT2 and HPT-1, at higher levels compared to biopsies of the gastrointestinal tract. Therefore, they concluded that Caco-2 cells may not reflect actual expression in different regions of the human gastrointestinal tract. Tai et al. [48] confirmed the presence of PEPT1, PHT1, PHT2 and PEPT2 in their Caco-2 cells at the RNA level and PEPT1, PHT1 and PEPT2 at the protein level. Interestingly, PEPT2 expression does not occur in the human intestine. Hence, when using Caco-2 cells, this might result in an overestimation of peptide absorption [49]. Others showed that PEPT1 expression only became apparent after 25 days of culturing [50]. This difference can be explained by inter-laboratories variations. Behrens et al. [51] studied the effect of cultivation duration, type of membrane support, seeding density, medium supplement and cell supplier on PEPT1 and HPT-1 expression in Caco-2 cells. They showed that the PEPT1 and HPT-1 expression reached a maximum plateau after three to four weeks in culture. Different materials of membrane support did not impact the peptide transporter expression, though collagen coating increased expression levels. Culture density played no role in PEPT1 expression, whereas it did for HPT-1 expression, and increasing substrates in the medium caused an increase in transporter expression [51]. In addition, cells from different suppliers showed variations in expression levels in peptide transporters, from constant expression to no expression at all [52]. Furthermore, extensive passaging did affect the transport characteristics of Caco-2 cells, as high passage numbers showed increased transcellular permeability and reduced paracellular permeability and carrier-mediated transport [53]. This stresses the variety in expression levels in cells, which can occur during cultivation or even before.

Besides the expression of the peptide transporters, the expression of the Na^+^/H^+^ exchanger NHE3 has been reported to be stably expressed in the Caco-2 cells [54], suggesting that Caco-2 cells are well equipped to maintain a proper proton gradient. Last but not least, the functionality of the expressed transporters was shown via substrate transport assays for dipeptides (glycine-proline, glycine-leucine and glycine-sarcosine) and a tripeptide (valine-proline-proline) by showing transport over the Caco-2 monolayer [55,56,57].

### 5.2. Caco-2: Amino Acid Transporter Expression

Transport studies with amino acids are challenging because of the redundancy among the various transporters. Caco-2 cells have been shown to be able to apically absorb alanine [58,59,60], arginine [60,61], glutamine [62,63], glycine [64], histidine [48], proline [65], and taurine [66], and to apically absorb and basolaterally secrete alanine [67,68,69], lysine [70], phenylalanine [71,72], proline [73], taurine [69], aspartate and glutamate [74]. Via RT-PCR and/or Northern blotting techniques, expression of the apical amino acid transporter systems has been investigated by multiple groups: ASC [75], b^0,+^ (rBAT) [76], β [69], PAT [64,69], N (SN2) [76], X^−^_AG_ [76]. Similarly, the basolateral amino acid systems have been identified in Caco-2 cells as well: GlyT [77], y^+^L [76], L [76], y^+^ [61] and X_c_^−^ [76,78]. 4F2hc is shown to be present in the Caco-2 cells, although expressed to a greater extent compared to the human duodenum [79]. This, most likely, results from the origin of the cells as 4F2hc is known to be up-regulated in cancer [32].

To study transporter mechanisms, the ion content can be changed as ion-dependency differs between transporters. However, reports published contradictive conclusions. For example, the Na^+^-dependent and Na^+^-independent transport of phenylalanine by Hidalgo et al. [71] and Berger et al. [71,80], which would point to different transporters, viz., B^0,+^ and b^0,+^, respectively. Via transport studies, it has been concluded that Caco-2 cells express the apical amino acid transporter systems B^0^, B^0,+^, b^0,+^, β, PAT, and the basolateral amino acid transporter systems GlyT and L [57,58,59,60,61,62,63,64,65,66,67,68,69,70,71,72,73,74]. Nicklin et al. [65] reported the absence of the IMINO system.

Furthermore, Kekuda et al. [69,75] reported the presence of ASC system-associated ATB^0,+^, whereas Anderson et al. [69] reported ATB^0,+^ to be below the detection limit. To our knowledge, there is no literature showing the presence of the basolateral amino acid systems A and Asc in Caco-2 cells. Caco-2 cells have proven suitability to study amino acid transport and express most of the relevant transporters. However, differences between laboratories cannot be ignored and should be taken into account when Caco-2 cells are used. Confirming the presence or absence of the desired transporters is strongly advised and is necessary for data interpretation when studying (novel) protein digestive absorption.

## 6. HT-29

The most abundant intestinal peptide transporters within the human intestinal tract, PEPT1 and PHT2, are absent in parent HT-29 cells [62,76,81], whereas PEPT2 and PHT1 are present [81]. HT-29 parent cells are highly sensitive to changes in culture medium. Lindley et al. [82] investigated the effects of different media and demonstrated that culturing in galactose-rich medium resulted in the expression of all four transporters. HT-29 cells are capable of absorbing taurine at a 5-fold higher activity compared to Caco-2 cells, whereas β-alanine was transported at similar rates. This indicates that the system β is present in these cells [83,84]. Via gene expression analysis and immunostaining, the presence of apical transporter systems N (SN2), b^0,+^ (rBAT), ASC and X^−^_AG_ and the basolateral transporter systems L (LAT2), X_c_^−^ and y^+^L has been reported [76,85]. However, Oda et al. [86] were unable to stain for LAT2, whereas staining for the basolateral-associated subunit 4F2hc was successful. By contrast, Kekuda et al. [76,85] and Bourgine et al. [76] did show the presence of LAT2.

HT-29/MTX cells are capable of transporting the dipeptide (Gly-Pro) at a similar magnitude as Caco-2 monolayers, proving the expression of apical peptide transporters [87]. However, to our knowledge, there is no specification as to which intestinal peptide transporters are present in HT29/MTX cells. HT29/MTX cells are capable of transporting phenylalanine, although the rate is lower compared to Caco-2 cells. Therefore, Hilgendorf et al. [87] postulated that the relevant amino acid transporters had lower expression levels.

## 7. T84

Little is known about apical and basolateral peptide and amino acid transporter expression in the T84 cell line. Merlin et al. [88] showed the absence of PEPT1 in the T84 cell line via RT-PCR. By contrast, Bourgine et al. [76] showed the expression of the peptide transporter PEPT1. Furthermore, the apical amino acid transporter systems N and X^−^_AG_ were described alongside the basolateral systems X_c_^−^, y^+^L and L.

**Figure 1:**
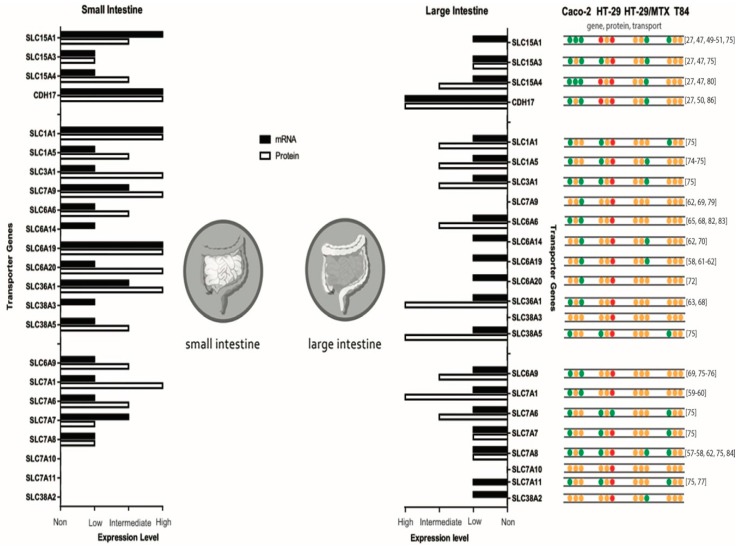
Summary of mRNA and protein expression levels of di-, tripeptide and amino acid transporters in the small and large intestine and gathered data of Caco-2, HT-29, HT-29/MTX and T84 cell lines. Protein expression in the small intestine of *SLC7A10* and *SLC7A11* and in the large intestine of *SLC7A10*, *SLC7A11* and *SLC38A3* is unknown. Expression levels originate from proteinatlas.org. For the gathered cell line data, green dots refer to presence, red dots refer to absent and orange dots refer to an unknown situation for that specific transporter. Caco-2 [27,47,49,50,51,57,58,59,60,61,62,63,65,68,69,70,72,74,83,84,86], HT-29 [70,75,76,77,78,79,80], HT-29/MTX [82], and T84 [70,82].

Figure 1 shows a comparison of the human expression at the gene and protein level of both the small and large intestine. In addition, an overview of transporter expressions in the described cell lines is shown.

## 8. Contradictive Results Due to Inter-Laboratory Differences

Within the field of cellular research, inter-laboratory differences are a well-known challenge. As described, di- and tripeptide and amino acid transporter expression levels are no exception for these inter-laboratorial differences. Harmonizing culture conditions between laboratories is cumbersome as each laboratory has developed its own culture protocol which has proven to be successful. Therefore, it is important to understand that the presence and/or abundances of e.g., the di- and tripeptide and amino acid transporters may vary in another laboratory. Thus, the importance of showing the presence of di- and tripeptides and amino acid transporters in your cell line is stressed when utilizing them, for e.g., protein absorption. Inter-laboratory studies are considered to harmonize and improve overall data interpretation. This was demonstrated by Zucco et al. [89], who studied intestinal barrier function and Hayeshi et al. [90], who focused on differences in drug and nutrient transporter expression in Caco-2 cells between different laboratories. However, to our knowledge, inter-laboratory studies have not been performed focusing on peptide and amino acid transporters

## 9. Advanced Culturing Conditions and Future Perspectives

While studying di- and tripeptide and amino acid absorption in cell models, it is important to have a model that is as physiological relevant as possible, especially when such a model is being used as screening tool for novel proteins. Showing the presence of a functional barrier, brush border enzymes and di- and tripeptide and amino acid transporters is considered to be essential—not only from the absorption point of view—as cell models are considered a relevant tool to screen for health effects as well. Commonly studied topics are focused on the prevention and recovery of barrier disruption [91]. Furthermore, cell models are a useful tool to study molecular pathways, such as the amino acid sensing pathway. Caco-2 and HT-29 cells have been shown to be responsive to changes in amino acid availability via the amino acid response pathway or its associated genes [92,93,94,95,96]. Unfortunately, to our knowledge, there is no literature directly connecting amino acid availability to specific peptide and amino acid transporter expression in the cell lines described. Closing this gap of knowledge will provide opportunities to optimize culture conditions by regulating transporter expression. Overall, availability of physiological relevant intestinal models will improve screening methods for novel nutrients and drug candidates.

In an attempt to improve the physiological relevance of cell lines, co-cultures of different epithelial cell lines have been proposed to provide a better representation of the small intestine. Verhoeckx et al. [97] concluded that a co-culture of Caco-2 and HT-29 provided a more physiological relevant cell population compared to monocultures. This is because the Caco-2 cells provide the barrier function and absorptive enterocyte population, whereas the HT-29 cells present the mucus producing goblet cells. Unfortunately, different growth rates between the cell lines result in HT-29 overgrowth, which could be partially overcome by adapting their ratios. Pan et al. [98] concluded that a 9:1 ratio of Caco-2:HT-29 is ideal. In line with this way of thinking, Hidalgo et al. [87] performed a transport study using the amino acid phenylalanine and dipeptide glycine-proline over a Caco-2:HT-29/MTX co-culture at different ratios. Phenylalanine transport decreased as the amount of HT-29/MTX in the co-culture increased, whereas dipeptide transport was not affected compared to the Caco-2 monoculture. These collective data suggest that the attempt to obtain a heterogeneous cell population was successful. However, it did affect the actual absorption, and the model needs constant adaptations as cell growth changes after cell passaging.

Kim et al. [99] developed a more complex in vitro system with Caco-2 cells in which the cells were positively stained for mucin 2, present in goblet cells. Research groups are developing 3-dimensional culturing systems. These models combine more physiological relevant parameters such as shear stress and microbiota [99,100]. Kim et al. [99,101] established a model mimicking the peristaltic movement of the intestine, through which they concluded that addition of flow altered the rate of differentiation and spontaneous formation of villi. Furthermore, they introduced microbial co-cultures on fully differentiated Caco-2 cells, while leaving the cells viable. Shah et al. [100] developed a microfluidic device, bringing together flow and microbial interaction. Just like Kim et al. [99,101], they concluded that cells can be co-cultured with microbial compounds in a controlled manner. Another innovative model, recently developed by Trietsch et al., consists of 3-dimensional tube-like structures of Caco-2 cells that form a tight barrier without the presence of a membrane and has proven to be a suitable tool to screen for effects on the intestinal barrier in a high-throughput manner [102]. These microfluidic devices will most likely become widely accepted, as they are easy to reproduce and are economically attractive [103,104]. As these novel models are still in the developmental stage, the effect of these alterations on the presence of peptide and amino acid transporters has yet to be determined.

The next step is combining different organs in the same system, thereby enabling the possibility to study organ cross-talk. The development of multi-organ platforms is accompanied by biological and technical challenges such as balancing in vivo complexity with simplicity, scaling of organs and inter-compartment sealing [105]. After nutrient absorption by the enterocytes, the liver may be the first organ which comes in contact with these compounds [106]. In addition, Peyer’s Patches may be encountered, facilitating the generation of the immune response within the mucosa. Though, combining the immune system with a gut-microfluidics platform is an ongoing challenge to address. Therefore, systems mimicking the gut-liver axis will have great value to study the steps after absorption. An example of such a model was developed by Choe et al. In their model, Caco-2 cells and HepG2 cells were co-cultured, and cell activity, intestinal barrier formation and the first pass metabolism of the drug Apigenin was assessed. They concluded that the cells could successfully be co-cultured; however, metabolic activity was shown to be enhanced in both cell types by evaluating P450 enzyme family activity, and the absorptive properties of Caco-2 cells changed as cell junctions were tighter and the absorptive permeability decreased. Furthermore, they concluded that their gut-liver-model better resembled the in vivo pharmacokinetics of Apigenin compared to a gut-monoculture [107]. Similar to the above-mentioned microfluidic intestinal models, these models are still in the developmental stage, and peptide and amino acid transporters have, as of yet, not been investigated.

Besides the effect of flow as introduced by microfluidic devices, the role of the extracellular matrix providing a 3-dimensional cell culturing is being widely studied. For this, hydrogels are highly suitable, hydrophilic polymeric materials that swell in contact with water and, depending on the proteins incorporated, provide a stiff material suitable for 3-dimensional cell growth. Most often studied in this regard is Matrigel^®^, which is composed of basement membrane proteins that are cytocompatible and can be formed into desired structures [108]. Addition of an extracellular matrix was shown to improve culture conditions supportive for maintaining proliferation and differentiation [109]. This allows cells to develop into mini-guts, also call organoids, as was demonstrated by McCracken et al. [110], who cultured stem cells on Matrigel^®^-coated wells and showed differentiation towards a small human intestine phenotype [111]. Addition of an extracellular matrix in non-organoid culture showed improvement in transporter expression levels in Caco-2 cells [51], suggesting the added value of the matrix in performing protein absorption assays.

In the future, physiologically improved cell culture models will have great potential for a broad range of applications. We foresee that in the future, organoid cultures will play a role in personalized medicine and nutritional intervention. Within the same time frame, more complex microfluidic devices are being developed using traditional cell lines e.g., Caco-2 cells. As the Caco-2 cells are well-defined, effects of changes in the micro-environment (e.g., 3D-culture, shear stress) on parameters (e.g., cell differentiation and barrier formation) can be effectively studied using these cells. However, the development of these novel 3-dimensional culture models is still in its infancy, and the effect on peptide and amino acid transporters has yet to be investigated in more detail. Besides cell differentiation, the microbiota play an important role in the health of an individual. We expect an interplay between organoid cultures, personal microbiota and microfluidic devices in the near future. As more complex culture systems are currently being developed, we stress the importance of an extensive description of all characteristics that researchers can think of when performing experiments with intestinal epithelial cells, in order to bring the field forward.

The final hurdle is the in vivo extrapolation of experimentally obtained data, for which different options can be considered. Often, in vitro results cannot be directly applied to predict human responses; therefore, it is important to build a consistent and reliable in vitro to in vivo extrapolation method. Such method uses, for example, mathematical modeling as shown by Fredlund et al. [112] or an actual comparison between in vitro and in vivo data as shown by Lennernäs et al. [113]. Yet, most knowledge is obtained from drug disposition studies, whereas a large gap still exists for nutrient absorption although proteins have been studied as drug delivery systems, such as casein [114]. Eventually, the integration of information gained from complex in vitro models into computational algorithms that incorporate human-specific physiological parameters will facilitate production of sustainable, novel nutrients.

## Figures and Tables

**Table 1:** Characteristics of diverse di- and tripeptide and amino acid transporters expressed in human intestine.

	Encoding Gene	Transporter Protein	Transporter System	Mechanism	Ion Dependency	Dimer Formation	Substrate
Di- and tripeptide transporters							
Apical membrane	*SLC15A1*	PEPT1		S	H^+^		Di- and tripeptide
	*SLC15A3*	PHT2		S	H^+^		Histidine, di- and tripeptide
	*SLC15A4*	PHT1		S	H^+^		Histidine, di- and tripeptide
	*CDH17*	HPT-1		S	H^+^		Di- and tripeptide
Basolateral membrane		Basolateral peptide transporter		U			Di- and tripeptide
Amino acid transporters							
Apical membrane	*SLC1A1*	EAAT3/EAAC1	X^−^_AG_	A	AA + 3Na^+^ + H^+^ ↔ K^+^		Aspartic acid and glutamic acid
	*SLC1A5*	ASCT2/AAAT	ASC	A	Na^+^ + AA ↔ Na^+^ + AA		Neutral amino acids primary substrates: alanine, asparagine, cysteine, glutamine, serine and threonine
	*SLC7A9*	b^0,+^AT	b^0,+^	A	CAA/cystine ↔ NAA	rBAT	Cationic amino acids and cystine
	*SLC6A6*	TauT	β	S	Cl^−^ and 2 Na^+^		β-alanine and taurine
	*SLC6A14*	ATB^0,+^	B^0,+^	S	2 Cl^−^ and Na^+^		Cationic and neutral amino acids
	*SLC6A19*	B^0^AT1/HND	B^0^	S	Na^+^		Neutral amino acids
	*SLC6A20*	SIT1	IMINO	S	Cl^−^ and 2Na^+^		Proline
	*SLC36A1*	PAT1/LYAAT1	PAT	S	H^+^		β-alanine, glycine and proline
	*SLC38A3*	SN1/SNAT3	N	A	AA + Na^+^ ↔ H^+^		Alanine, asparagine, glutamine and histidine
	*SLC38A5*	SN2/SNAT5	N	A	AA + Na^+^ ↔ H^+^		Asparagine, glutamine, histidine and serine
Basolateral membrane	*SLC6A9*	GlyT1	Gly^+^	S	Cl^−^ and 2Na^+^		Glycine
	*SLC7A1*	CAT1	y^+^	U			Arginine, histidine and lysine
	*SLC7A6*	y^+^LAT2	y^+^L	A	CAA ↔ NAA + Na^+^	4F2hc	Cationic amino acids
	*SLC7A7*	y^+^LAT1	y^+^L	A	CAA ↔ NAA + Na^+^	4F2hc	Cationic amino acids
	*SLC7A8*	LAT2	L	A	NAA ↔ NAA	4F2hc	Neutral amino acids
	*SLC7A10*	asc-1	Asc	A	NAA ↔ NAA	4F2hc	Small neutral amino acids
	*SLC7A11*	xCT	X_c_	A	Cystine ↔ Glutamic acid	4F2hc	Cystine
	*SLC38A2*	SNAT2	A	S	Na^+^		Neutral amino acids and imino

At the apical membrane there are four peptide transporters expressed by *SLC15A1*, *SLC15A3*, *SLC15A4* and *CDH17* and one on the basolateral membrane with an unknown identity. There are nine apical amino acid transporter systems; *SLC1A1* (system X^−^_AG_), *SLC1A5* (system ASC), *SLC3A1* and *SLC7A9* (system b^0,+^), *SLC6A6* (system β), *SLC6A14* (system B^0,+^), *SLC6A19* (system B^0^), *SLC6A20* (system IMINO), *SLC36A1* (system PAT), *SLC38A3* and *SLC38A5* (system N). At the basolateral membrane, seven amino acid transporter systems can be distinguished; *SLC6A9* (system Gly), *SLC7A1* (system y^+^), *SLC7A6* and *SLC7A7* (system y^+^L), *SLC7A8* (system L), *SLC7A10* (system Asc), *SLC7A11* (system X_C_) and *SLC38A2* (system A). Amino acid transporters are often referred to by their transporter system in the literature. Mechanisms are depicted by S = symporter, U = uniporter and A = antiporter, as most of them have ion dependency of the required ions [27,30,37,38,39,40,41,42].

## References

[1] Cordain L., Eaton S.B., Sebastian A., Mann N., Lindeberg S., Watkins B.A., O’Keefe J.H., Brand-Miller J. (2005). Origins and evolution of the Western diet: Health implications for the 21st century. Am. J. Clin. Nutr..

[2] Frayn K.N. (2009). Metabolic Regulation: A Human Perspective.

[3] Nishizuka Y. (1984). The role of protein kinase C in cell surface signal transduction and tumour promotion. Nature.

[4] Hur S.J., Lim B.O., Decker E.A., McClements D.J. (2011). In vitro human digestion models for food applications. Food Chem..

[5] Berg J.M., Tymoczko J.L., Stryer L. (2002). Proteins Are Degraded to Amino Acids. Biochemistry.

[6] Raufman J.-P. (1992). Gastric chief cells: Receptors and signal-transduction mechanisms. Gastroenterology.

[7] Ehrhardt C., Kim K.-J. (2007). Drug Absorption Studies: In Situ, In Vitro and In Silico Models.

[8] Li H., Limenitakis J.P., Fuhrer T., Geuking M.B., Lawson M.A., Wyss M., Brugiroux S., Keller I., Macpherson J.A., Rupp S. (2015). The outer mucus layer hosts a distinct intestinal microbial niche. Nat. Commun..

[9] Nakamura N., Lin H.C., McSweeney C.S., Mackie R.I., Gaskins H.R. (2010). Mechanisms of microbial hydrogen disposal in the human colon and implications for health and disease. Annu. Rev. Food Sci. Technol..

[10] Turner J.R. (2016). Epithelia and Gastrointestinal Function. Yamada’s Textbook of Gastroenterology.

[11] Bornhorst G.M., Paul Singh R. (2014). Gastric digestion in vivo and in vitro: How the structural aspects of food influence the digestion process. Annu. Rev. Food Sci. Technol..

[12] Minekus M., Alminger M., Alvito P., Ballance S., Bohn T., Bourlieu C., Carrière F., Boutrou R., Corredig M., Dupont D. (2014). A standardised static in vitro digestion method suitable for food—An international consensus. Food Funct..

[13] Rinderknecht H. (1986). Activation of pancreatic zymogens. Dig. Dis. Sci..

[14] Erickson R.H., Kim Y.S. (1990). Digestion and absorption of dietary protein. Annu. Rev. Med..

[15] Neis E.P., Dejong C.H., Rensen S.S. (2015). The role of microbial amino acid metabolism in host metabolism. Nutrients.

[16] El Aidy S., Van Den Bogert B., Kleerebezem M. (2015). The small intestine microbiota, nutritional modulation and relevance for health. Curr. Opin. Biotechnol..

[17] Dai Z.-L., Wu G., Zhu W.-Y. (2011). Amino acid metabolism in intestinal bacteria: Links between gut ecology and host health. Front. Biosci..

[18] Jandhyala S.M., Talukdar R., Subramanyam C., Vuyyuru H., Sasikala M., Reddy D.N. (2015). Role of the normal gut microbiota. World J. Gastroenterol. WJG.

[19] Regazzo D., Mollé D., Gabai G., Tomé D., Dupont D., Leonil J., Boutrou R. (2010). The (193–209) 17-residues peptide of bovine β-casein is transported through Caco-2 monolayer. Mol. Nutr. Food Res..

[20] Terada T., Sawada K., Saito H., Hashimoto Y., Inui K.-I. (1999). Functional characteristics of basolateral peptide transporter in the human intestinal cell line Caco-2. Am. J. Physiol.-Gastrointest. Liver Physiol..

[21] Shimizu M. (1999). Modulation of intestinal functions by food substances. Mol. Nutr. Food Res..

[22] Ulluwishewa D., Anderson R.C., McNabb W.C., Moughan P.J., Wells J.M., Roy N.C. (2011). Regulation of tight junction permeability by intestinal bacteria and dietary components. J. Nutr..

[23] Alpers D.H., Taylor B., Klein S., Tadataka Y. (2008). General nutritional principles. Principles of Clinical Gastroenterology.

[24] Broer S. (2008). Amino acid transport across mammalian intestinal and renal epithelia. Physiol. Rev..

[25] Brandsch M., Knütter I., Leibach F.H. (2004). The intestinal H+/peptide symporter PEPT1: Structure–affinity relationships. Eur. J. Pharm. Sci..

[26] Dantzig A.H., Hoskins J., Tabas L.B., Bright S., Shepard R.L., Jenkins I.L., Duckworth D.C., Sportsman J.R., Mackensen D., Rosteck P.R. (1994). Association of intestinal peptide transport with a protein related to the cadherin superfamily. Science.

[27] Herrera-Ruiz D., Wang Q., Cook T.J., Knipp G.T., Gudmundsson O.S., Smith R.L., Faria T.N. (2001). Spatial expression patterns of peptide transporters in the human and rat gastrointestinal tracts, Caco-2 in vitro cell culture model, and multiple human tissues. AAPS Pharmsci..

[28] Rubio-Aliaga I., Daniel H. (2008). Peptide transporters and their roles in physiological processes and drug disposition. Xenobiotica.

[29] Adibi S.A., Morse E.L. (1971). Intestinal transport of dipeptides in man: Relative importance of hydrolysis and intact absorption. J. Clin. Investig..

[30] Kiela P.R., Ghishan F.K. (2016). Physiology of Intestinal Absorption and Secretion. Best Pract. Res. Clin. Gastroenterol..

[31] Barrett K.E., Keely S.J. (2016). Electrolyte Secretion and Absorption in the Small Intestine and Colon. Yamada's Textbook of Gastroenterology.

[32] Palacín M., Kanai Y. (2004). The ancillary proteins of HATs: SLC3 family of amino acid transporters. Pflüg. Arch..

[33] Fort J., Laura R., Burghardt H.E., Ferrer-Costa C., Turnay J., Ferrer-Orta C., Usón I., Zorzano A., Fernández-Recio J., Orozco M. (2007). The structure of human 4F2hc ectodomain provides a model for homodimerization and electrostatic interaction with plasma membrane. J. Biol. Chem..

[34] Fotiadis D., Kanai Y., Palacín M. (2013). The SLC3 and SLC7 families of amino acid transporters. Mol. Asp. Med..

[35] Irie M., Terada T., Okuda M., Inui K.-I. (2004). Efflux properties of basolateral peptide transporter in human intestinal cell line Caco-2. Pflüg. Arch..

[36] Kanai Y., Clémençon B., Simonin A., Leuenberger M., Lochner M., Weisstanner M., Hediger M.A. (2013). The SLC1 high-affinity glutamate and neutral amino acid transporter family. Mol. Asp. Med..

[37] Pramod A.B., Foster J., Carvelli L., Henry L.K. (2013). SLC6 transporters: Structure, function, regulation, disease association and therapeutics. Mol. Asp. Med..

[38] Schiöth H.B., Roshanbin S., Hägglund M.G.A., Fredriksson R. (2013). Evolutionary origin of amino acid transporter families SLC32, SLC36 and SLC38 and physiological, pathological and therapeutic aspects. Mol. Asp. Med..

[39] Terada T., Sawada K., Irie M., Saito H., Hashimoto Y., Inui K. (2000). Structural requirements for determining the substrate affinity of peptide transporters PEPT1 and PEPT2. Pflüg. Arch..

[40] Geiss-Friedlander R., Parmentier N., Moller U., Urlaub H., Van den Eynde B.J., Melchior F. (2009). The cytoplasmic peptidase DPP9 is rate-limiting for degradation of proline-containing peptides. J. Biol. Chem..

[41] Jewell J.L., Russell R.C., Guan K.-L. (2013). Amino acid signalling upstream of mTOR. Nat. Rev. Mol. Cell Biol..

[42] Taylor P.M. (2014). Role of amino acid transporters in amino acid sensing. Am. J. Clin. Nutr..

[43] Meunier V., Bourrie M., Berger Y., Fabre G. (1995). The human intestinal epithelial cell line Caco-2; pharmacological and pharmacokinetic applications. Cell Biol. Toxicol..

[44] Zweibaum A., Pinto M., Chevalier G., Dussaulx E., Triadou N., Lacroix B., Haffen K., Brun J.-L., Rousset M. (1985). Enterocytic differentiation of a subpopulation of the human colon tumor cell line HT-29 selected for growth in sugar-free medium and its inhibition by glucose. J. Cell. Physiol..

[45] Rousset M. (1986). The human colon carcinoma cell lines HT-29 and Caco-2: Two in vitro models for the study of intestinal differentiation. Biochimie.

[46] Lesuffleur T., Barbat A., Dussaulx E., Zweibaum A. (1990). Growth adaptation to methotrexate of HT-29 human colon carcinoma cells is associated with their ability to differentiate into columnar absorptive and mucus-secreting cells. Cancer Res..

[47] Madara J.L., Stafford J., Dharmsathaphorn K., Carlson S. (1987). Structural analysis of a human intestinal epithelial cell line. Gastroenterology.

[48] Tai W., Chen Z., Cheng K. (2012). Expression profile and functional activity of peptide transporters in prostate cancer cells. Mol. Pharm..

[49] Groneberg D.A., Doring F., Theis S., Nickolaus M., Fischer A., Daniel H. (2002). Peptide transport in the mammary gland: Expression and distribution of PEPT2 mRNA and protein. Am. J. Physiol. Endocrinol. Metab..

[50] Sambuy Y., De Angelis I., Ranaldi G., Scarino M.L., Stammati A., Zucco F. (2005). The Caco-2 cell line as a model of the intestinal barrier: Influence of cell and culture-related factors on Caco-2 cell functional characteristics. Cell Biol. Toxicol..

[51] Behrens I., Kissel T. (2003). Do cell culture conditions influence the carrier-mediated transport of peptides in Caco-2 cell monolayers?. Eur. J. Pharm. Sci..

[52] Behrens I., Kamm W., Dantzig A.H., Kissel T. (2004). Variation of peptide transporter (PepT1 and HPT1) expression in Caco-2 cells as a function of cell origin. J. Pharm. Sci..

[53] Yu H., Cook T.J., Sinko P.J. (1997). Evidence for diminished functional expression of intestinal transporters in Caco-2 cell monolayers at high passages. Pharm. Res..

[54] Tse C.M., Levine S.A., Yun C.H., Brant S.R., Pouyssegur J., Montrose M.H., Donowitz M. (1993). Functional characteristics of a cloned epithelial Na+/H+ exchanger (NHE3): Resistance to amiloride and inhibition by protein kinase C. Proc. Natl. Acad. Sci. USA.

[55] Saito H., Inui K. (1993). Dipeptide transporters in apical and basolateral membranes of the human intestinal cell line Caco-2. Am. J. Physiol..

[56] Satake M., Enjoh M., Nakamura Y., Takano T., Kawamura Y., Arai S., Shimizu M. (2002). Transepithelial transport of the bioactive tripeptide, Val-Pro-Pro, in human intestinal Caco-2 cell monolayers. Biosci. Biotechnol. Biochem..

[57] Thwaites D.T., Brown C.D.A., Hirst B.H., Simmons N.L. (1993). H^+^-coupled dipeptide (glycylsarcosine) transport across apical and basal borders of human intestinal Caco-2 cell monolayers display distinctive characteristics. Biochim. Biophys. Acta (BBA)-Biomembr..

[58] Pan M., Souba W.W., Karinch A.M., Lin C.-M., Stevens B.R. (2002). Epidermal growth factor regulation of system L alanine transport in undifferentiated and differentiated intestinal Caco-2 cells. J. Gastrointest. Surg..

[59] Pan M., Souba W.W., Wolfgang C.L., Karinch A.M., Stevens B.R. (2002). Posttranslational alanine trans-stimulation of zwitterionic amino acid transport systems in human intestinal Caco-2 cells. J. Surg. Res..

[60] Pan M., Stevens B.R. (1995). Protein kinase C-dependent regulation of L-arginine transport activity in Caco-2 intestinal cells. Biochim. Biophys. Acta (BBA)-Biomembr..

[61] Pan M., Malandro M., Stevens B.R. (1995). Regulation of system y+ arginine transport capacity in differentiating human intestinal Caco-2 cells. Am. J. Physiol..

[62] Souba W.W., Copeland E.M. (1992). Cytokine modulation of Na(+)-dependent glutamine transport across the brush border membrane of monolayers of human intestinal Caco-2 cells. Ann. Surg..

[63] Costa C., Huneau J.-F., Tomé D. (2000). Characteristics of L-glutamine transport during Caco-2 cell differentiation. Biochim. Biophys. Acta (BBA)-Biomembr..

[64] Chen Z., Fei Y.J., Anderson C.M., Wake K.A., Miyauchi S., Huang W., Thwaites D.T., Ganapathy V. (2003). Structure, function and immunolocalization of a proton-coupled amino acid transporter (hPAT1) in the human intestinal cell line Caco-2. J. Physiol..

[65] Nicklin P.L., Irwin W.J., Hassan I.F., Mackay M. (1992). Proline uptake by monolayers of human intestinal absorptive (Caco-2) cells in vitro. Biochim. Biophys. Acta (BBA)-Biomembr..

[66] Satsu H., Watanabe H., Arai S., Shimizu M. (1997). Characterization and regulation of taurine transport in Caco-2, human intestinal cells. J. Biochem..

[67] Thwaites D.T., McEwan G.T., Brown C.D., Hirst B.H., Simmons N.L. (1993). Na(+)-independent, H(+)-coupled transepithelial beta-alanine absorption by human intestinal Caco-2 cell monolayers. J. Biol. Chem..

[68] Thwaites D.T., McEwan G.T.A., Brown C.D.A., Hirst B.H., Simmons N.L. (1994). L-Alanine absorption in human intestinal Caco-2 cells driven by the proton electrochemical gradient. J. Membr. Biol..

[69] Anderson C.M.H., Howard A., Walters J.R.F., Ganapathy V., Thwaites D.T. (2009). Taurine uptake across the human intestinal brush-border membrane is via two transporters: H^+^-coupled PAT1 (SLC36A1) and Na^+^-and Cl^−^-dependent TauT (SLC6A6). J. Physiol..

[70] Thwaites D.T., Markovich D., Murer H., Simmons N.L. (1996). Na^+^-independent lysine transport in human intestinal Caco-2 cells. J. Membr. Biol..

[71] Hidalgo I.J., Borchardt R.T. (1990). Transport of a large neutral amino acid (phenylalanine) in a human intestinal epithelial cell line: Caco-2. Biochim. Biophys. Acta (BBA)-Biomembr..

[72] Hu M., Borchardt R.T. (1992). Transport of a large neutral amino acid in a human intestinal epithelial cell line (Caco-2): Uptake and efflux of phenylalanine. Biochim. Biophys. Acta (BBA)-Mol. Cell Res..

[73] Thwaites D.T., McEwan G.T.A., Cook M.J., Hirst B.H., Simmons N.L. (1993). H^+^-coupled (Na^+^-independent) proline transport in human intestinal (Caco-2) epithelial cell monolayers. FEBS Lett..

[74] Nicklin P.L., Irwin W.J., Hassan I.F., Mackay M., Dixon H.B.F. (1995). The transport of acidic amino acids and their analogues across monolayers of human intestinal absorptive (Caco-2) cells in vitro. Biochim. Biophys. Acta (BBA)-Mol. Cell Res..

[75] Kekuda R., Torres-Zamorano V., Fei Y.J., Prasad P.D., Li H.W., Mader L.D., Leibach F.H., Ganapathy V. (1997). Molecular and functional characterization of intestinal Na(+)-dependent neutral amino acid transporter B0. Am. J. Physiol..

[76] Bourgine J., Billaut-Laden I., Happillon M., Lo-Guidice J.M., Maunoury V., Imbenotte M., Broly F. (2012). Gene expression profiling of systems involved in the metabolism and the disposition of xenobiotics: Comparison between human intestinal biopsy samples and colon cell lines. Drug Metab. Dispos..

[77] Christie G.R., Ford D., Howard A., Clark M.A., Hirst B.H. (2001). Glycine supply to human enterocytes mediated by high-affinity basolateral GLYT1. Gastroenterology.

[78] Bassi M., Gasol E., Manzoni M., Pineda M., Riboni M., Martín R., Zorzano A., Borsani G., Palacín M. (2001). Identification and characterisation of human xCT that co-expresses, with 4F2 heavy chain, the amino acid transport activity system xc–. Pflüg. Arch..

[79] Sun D., Lennernas H., Welage L.S., Barnett J.L., Landowski C.P., Foster D., Fleisher D., Lee K.-D., Amidon G.L. (2002). Comparison of human duodenum and Caco-2 gene expression profiles for 12,000 gene sequences tags and correlation with permeability of 26 drugs. Pharm. Res..

[80] Berger V., Larondelle Y., Trouet A., Schneider Y.J. (2000). Transport mechanisms of the large neutral amino acid l-phenylalanine in the human intestinal epithelial caco-2 cell line. J. Nutr..

[81] Liang R., Fei Y.J., Prasad P.D., Ramamoorthy S., Han H., Yang-Feng T.L., Hediger M.A., Ganapathy V., Leibach F.H. (1995). Human intestinal H+/peptide cotransporter. Cloning, functional expression, and chromosomal localization. J. Biol. Chem..

[82] Lindley D.J., Roth W.J., Carl S.M., Knipp G.T. (2012). The effects of media on pharmaceutically relevant transporters in the human HT-29 adenocarcinoma cell line: Does culture media need to be controlled?. J. Pharm. Sci..

[83] Tiruppathi C., Brandsch M., Miyamoto Y., Ganapathy V., Leibach F.H. (1992). Constitutive expression of the taurine transporter in a human colon carcinoma cell line. Am. J. Physiol..

[84] Brandsch M., Miyamoto Y., Ganapathy V., Leibach F.H. (1993). Regulation of taurine transport in human colon carcinoma cell lines (HT-29 and Caco-2) by protein kinase C. Am. J. Physiol..

[85] Kekuda R., Prasad P.D., Fei Y.-J., Torres-Zamorano V., Sinha S., Yang-Feng T.L., Leibach F.H., Ganapathy V. (1996). Cloning of the sodium-dependent, broad-scope, neutral amino acid transporter Bo from a human placental choriocarcinoma cell line. J. Biol. Chem..

[86] Oda K., Hosoda N., Endo H., Saito K., Tsujihara K., Yamamura M., Sakata T., Anzai N., Wempe M.F., Kanai Y. (2010). L-type amino acid transporter 1 inhibitors inhibit tumor cell growth. Cancer Sci..

[87] Hilgendorf C., Spahn-Langguth H., Regårdh C.G., Lipka E., Amidon G.L., Langguth P. (2000). Caco-2 versus caco-2/HT29-MTX co-cultured cell lines: Permeabilities via diffusion, inside-and outside-directed carrier-mediated transport. J. Pharm. Sci..

[88] Merlin D., Steel A., Gewirtz A.T., Si-Tahar M., Hediger M.A., Madara J.L. (1998). hPepT1-mediated epithelial transport of bacteria-derived chemotactic peptides enhances neutrophil-epithelial interactions. J. Clin. Investig..

[89] Zucco F., Batto A., Bises G., Chambaz J., Chiusolo A., Consalvo R., Cross H., Dal Negro G., de Angelis I., Fabre G. (2005). An inter-laboratory study to evaluate the effects of medium composition on the differentiation and barrier function of Caco-2 cell lines. ATLA-NOTTINGHAM-.

[90] Hayeshi R., Hilgendorf C., Artursson P., Augustijns P., Brodin B., Dehertogh P., Fisher K., Fossati L., Hovenkamp E., Korjamo T. (2008). Comparison of drug transporter gene expression and functionality in Caco-2 cells from 10 different laboratories. Eur. J. Pharm. Sci..

[91] Akbari P., Braber S., Gremmels H., Koelink P.J., Verheijden K.A., Garssen J., Fink-Gremmels J. (2014). Deoxynivalenol: A trigger for intestinal integrity breakdown. FASEB J..

[92] Bruhat A., Jousse C., Wang X.-Z., Ron D., Ferrara M., Fafournoux P. (1997). Amino acid limitation induces expression of CHOP, a CCAAT/enhancer binding protein-related gene, at both transcriptional and post-transcriptional levels. J. Biol. Chem..

[93] Le Bacquer O., Laboisse C., Darmaun D. (2003). Glutamine preserves protein synthesis and paracellular permeability in Caco-2 cells submitted to “luminal fasting”. Am. J. Physiol.-Gastrointest. Liver Physiol..

[94] Chaumontet C., Azzout-Marniche D., Gaudichon C., Gausserès N., Vinoy S., Tomé D. (2007). AMPK phosphorylation is decreased in response to amino acids and glucose in Caco-2 intestinal cells. FASEB J..

[95] Vynnytska-Myronovska B.O., Kurlishchuk Y., Chen O., Bobak Y., Dittfeld C., Hüther M., Kunz-Schughart L.A., Stasyk O.V. (2016). Arginine starvation in colorectal carcinoma cells: Sensing, impact on translation control and cell cycle distribution. Exp. Cell Res..

[96] Bobak Y., Kurlishchuk Y., Vynnytska-Myronovska B., Grydzuk O., Shuvayeva G., Redowicz M.J., Kunz-Schughart L.A., Stasyk O. (2016). Arginine deprivation induces endoplasmic reticulum stress in human solid cancer cells. Int. J. Biochem. Cell Biol..

[97] Verhoeckx K., Cotter P., López-Expósito I., Kleiveland C., Lea T., Mackie A., Requena T., Swiatecka D., Wichers H. (2015). The Impact of Food Bioactives on Health.

[98] Pan F., Han L., Zhang Y., Yu Y., Liu J. (2015). Optimization of Caco-2 and HT29 co-culture in vitro cell models for permeability studies. Int. J. Food Sci. Nutr..

[99] Kim H.J., Huh D., Hamilton G., Ingber D.E. (2012). Human gut-on-a-chip inhabited by microbial flora that experiences intestinal peristalsis-like motions and flow. Lab Chip.

[100] Shah P., Fritz J.V., Glaab E., Desai M.S., Greenhalgh K., Frachet A., Niegowska M., Estes M., Jäger C., Seguin-Devaux C. (2016). A microfluidics-based in vitro model of the gastrointestinal human-microbe interface. Nat. Commun..

[101] Kim H.J., Ingber D.E. (2013). Gut-on-a-Chip microenvironment induces human intestinal cells to undergo villus differentiation. Integr. Biol..

[102] Trietsch S.J., Naumovska E., Kurek D., Setyawati M.C., Vormann M.K., Wilschut K.J., Lanz H.L., Nicolas A., Ng C.P., Joore J. (2017). Membrane-free culture and real-time barrier integrity assessment of perfused intestinal epithelium tubes. Nat. Commun..

[103] Valencia P.M., Farokhzad O.C., Karnik R., Langer R. (2012). Microfluidic technologies for accelerating the clinical translation of nanoparticles. Nat. Nanotechnol..

[104] Li X.J., Zhou Y. (2013). Microfluidic Devices for Biomedical Applications.

[105] Rogal J., Probst C., Loskill P. (2017). Integration concepts for multi-organ chips: How to maintain flexibility?!. Future Sci. OA.

[106] Zeuzem S. (2000). Gut-liver axis. Int. J. Colorectal Dis..

[107] Choe A., Ha S.K., Choi I., Choi N., Sung J.H. (2017). Microfluidic Gut-liver chip for reproducing the first pass metabolism. Biomed. Microdevices.

[108] Kleinman H.K., Martin G.R. (2005). Matrigel: Basement membrane matrix with biological activity. Semin. Cancer Biol..

[109] Basson M.D., Turowski G., Emenaker N.J. (1996). Regulation of human (Caco-2) intestinal epithelial cell differentiation by extracellular matrix proteins. Exp. Cell Res..

[110] McCracken K.W., Howell J.C., Wells J.M., Spence J.R. (2011). Generating human intestinal tissue from pluripotent stem cells in vitro. Nat. Protoc..

[111] Watson C.L., Mahe M.M., Múnera J., Howell J.C., Sundaram N., Poling H.M., Schweitzer J.I., Vallance J.E., Mayhew C.N., Sun Y. (2014). An in vivo model of human small intestine using pluripotent stem cells. Nat. Med..

[112] Fredlund L., Winiwarter S., Hilgendorf C. (2017). In Vitro Intrinsic Permeability: A Transporter-Independent Measure of Caco-2 Cell Permeability in Drug Design and Development. Mol. Pharm..

[113] Lennernäs H., Palm K., Fagerholm U., Artursson P. (1996). Comparison between active and passive drug transport in human intestinal epithelial (Caco-2) cells in vitro and human jejunum in vivo. Int. J. Pharm..

[114] Bani-Jaber A., Alshawabkeh I., Abdullah S., Hamdan I., Ardakani A., Habash M. (2017). In Vitro and In Vivo Evaluation of Casein as a Drug Carrier for Enzymatically Triggered Dissolution Enhancement from Solid Dispersions. AAPS PharmSciTech.

